# Post-diagnostic antipsychotic use and cancer mortality: a population based cohort study

**DOI:** 10.1186/s12885-020-07320-3

**Published:** 2020-08-24

**Authors:** Blánaid M. Hicks, John Busby, Ken Mills, Francis A. O’Neil, Stuart A. McIntosh, Shu-Dong Zhang, Fabio Giuseppe Liberante, Chris R. Cardwell

**Affiliations:** 1 Centre for Public Health, ICSB, Royal Victoria Hospital, Belfast, BT12 6BA Northern Ireland; 2 Centre for Cancer Research and Cell Biology (CCRCB), Queen’s University Belfast, Belfast, Northern Ireland; 3 Breast Surgery Department, Belfast City Hospital, Belfast Health and Social Care Trust, Belfast, Northern Ireland, UK; 4 Northern Ireland Centre for Stratified Medicine, Biomedical Sciences Research Institute, University of Ulster, C-TRIC Building, Altnagelvin Area Hospital, Londonderry, UK; 5 Ludwig Boltzmann Institute of Cancer Research, Vienna, Austria

**Keywords:** Antipsychotics, Prolactin, Breast cancer, Survival

## Abstract

**Background:**

Many antipsychotics elevate prolactin, a hormone implicated in breast cancer aetiology however no studies have investigated antipsychotic use in patients with breast cancer. This study investigated if antipsychotic use is associated with an increased risk of cancer-specific mortality among breast cancer patients.

**Methods:**

A cohort of 23,695 women newly diagnosed with a primary breast cancer between 1st January 1998 and 31st December 2012 was identified from the UK Clinical Practice Research Datalink linked to English cancer-registries and followed for until 30th September 2015. Time-dependent Cox proportional hazards models were used to calculate adjusted hazard ratios (HRs) and 95% confidence intervals (CIs) of breast cancer-specific mortality comparing use of antipsychotics with non-use, overall, and by prolactin elevating activitiy. Analyses were repeated restricting to patients with a history of severe mental illness to control for potential confounding by indication.

**Results:**

In total 848 patients were prescribed an antipsychotic and of which 162 died due to their breast cancer. Compared with non-use, antipsychotic use was associated with an increased risk of breast-cancer specific mortality (HR 2.25, 95%CI 1.90–2.67), but this did not follow a dose response relation. Restricting the cohort to patients with severe mental illness attenuated the association between antipsychotic use and breast cancer-specific mortality (HR 1.11, 95%CI 0.58–2.14).

**Conclusions:**

In this population-based cohort of breast cancer patients, while the use of antipsychotics was associated with increased breast cancer-specific mortality, there was a lack of a dose response, and importantly null associations were observed in patients with severe mental illness, suggesting the observed association is likely a result of confounding by indication. This study provides an exemplar of confounding by indication, highlighting the importance of consideration of this important bias in studies of drug effects in cancer patients.

## Background

Antipsychotic medications are used in a range of clinical settings including in the first-line stetting for schizophrenia, other psychosis and bipolar disorder [1]. Recent evidence form UK general practice has also shown they are increasingly used for other indications including for example anxiety disorders, depression, persobality disorders and antention deficit hyperactivity disorder (ADHD) [1]. Yet prescribing rates vary enormously worldwide. The mechanism of action on psychosis is presumed to relate to their modulation of the brain’s dopaminergic system and in particular the blocking of Dopamine receptor D_2_ receptors (D2R) in the mesolimbic system. However they are a very heterogenous group of drugs with a range of actions and side effects.

A particularly common side effect of antipsychotics is an elevation of prolactin as a consequence of their direct effect of blocking D2R in the pituitary [2]. All antipsychotics may cause a temporary increase in prolactin release but some (including first-generation antipsychotics and some second-generation antipsychotics such as risperidone and amisulpride) have been shown to prolong elevation of prolactin levels leading to osteoporosis, galactorrhoea and sexual dysfunction [3, 4]. The potency of this effect on prolactin levels may be influenced not just by the ability of the drug to bind to D2 centrally but also by its ability to cross the blood-brain barrier (BBB) as the pituitary lies outside the BBB.

Prolactin is implicated in both breast cancer aetiology and progression. Studies show increased expression of prolactin receptors on breast cancer tissue and prolactin induced proliferation of breast cancer cells [5, 6]. Observational studies show that patients with higher plasma prolactin levels have increased risks of breast cancer and increased risks of breast cancer progression and mortality [7–10]. Although numerous observational studies of antipsychotic use and breast cancer report null associations [11–16], a recent Danish study, including 4951 breast cancer cases, found increases in the risk of oestrogen receptor positive breast cancer with long-term antipsychotic use [17].

Given this evidence, there are concerns about the safety of prescribing antipsychotics to breast cancer patients with mental illnesses. For example Rahman *et.al* recommended that several antipsychotics should be avoided in breast cancer patients and in the USA, supplementary package inserts contain warnings about using antipsychotics in breast cancer patients [18]. In contrast, researchers have argued that the published data linking prolactin to breast cancer risk and progression are unconvincing and insufficient to deprive breast cancer patients of antipsychotic treatment [19]. Despite this debate, no previous studies have investigated the association between antipsychotic use and breast cancer survival. Therefore, we aim to investigate whether post-diagnostic antipsychotic use increased mortality among a population-based cohort of breast cancer patients.

## Methods

### Data sources

This study was conducted using the UK Clinical Practice Research Datalink (CPRD), linked to English cancer registry data from the National Cancer Data Repository (NCDR), and death registration data from the Office for National Statistics (ONS). The CPRD contains data from 674 general practices, including more than 15 million patients, approximately 6.9% of the UK population, and has been shown to be representative [20]. The CPRD records information on demographics, anthropometric and lifestyle information, clinical diagnoses and prescription data. Previous research has found CPRD prescription and clinical information to be of high quality and validity [21, 22]. The CPRD are audited for data completeness and quality. Practices meeting a predefined quality standard are deemed ‘up to research standard’ and included in future data extracts. The NCDR holds UK-wide data from English cancer registries compiled from a variety of sources including general practices, cancer screening programmes, NHS and private hospitals, and death certificates [23]. ONS death-registration data provide details on the date and cause(s) of death.

CPRD obtains ethical approval to receive and supply patient data for public health research. The study protocol was approved by the Scientific Advisory Committee of the CPRD (protocol number 16_079R).

### Study population

A cohort of female patients with newly-diagnosed invasive breast cancer between 1998 and 2012, were identified from the NCDR. Patients with a previous record of cancer were identified and excluded from the analysis using a list of cancer Read codes modified for use in the CPRD [23]. Further exclusions included those patients diagnosed with a breast cancer before they were registered with a CPRD practice, before their practice was deemed up to research standard, after they left a CPRD practice, or after data was last collected from their practice by the CPRD.

Deaths were identified from ONS records. Breast cancer-specific deaths were defined as those with breast cancer (ICD-10 C50) recorded as the primary underlying cause of death. Patients who died within the first year of the study were excluded for latency considerations, as short exposure duration is unlikely associated with cancer survival. Thus, patients were followed from 1 year after breast cancer diagnosis (T0) through to the date of death, end of registration with the general practice, last collection of data from the practice, end of the study period (30th September 2015), whichever occurred first.

### Exposure definition

We considered all antipsychotics available in the UK, based on the British National Formulary (as listed in Supplementary Table S1) [24]. Prochlorperazine, Droperidol and levpromazine were not included to reduce confounding by indication, as these are also used as antiemetics (often used to eliviate nausa associated with chemotherpy or terminal illness) or indicated in palliative care. The use of post-diagnostic antipsychotics was considered as a time-varying variable in which each person-day was classified as either antipsychotic use or non-use, allowing patients to contribute both exposed and unexposed person-time to the analysis. The use of a time-varying exposure definition accounts for immortal time bias and has been recommended previously [25]. Exposure was lagged by 1 year to account for a biologically meaningful latency time window, given that short exposure duration is unlikely associated with cancer survival and to minimize reverse causality. Thus, patients were considered unexposed to antipsychotics until 1 year after their first antipsychotic prescription and considered exposed thereafter for the remainder of follow-up (as illustrated in Supplementary Fig. S1). To enable testing of dose-response relationships we extracted data on the medication prescribed, number of tablets and medication strength and calculated the defined daily dose (DDD) for each prescription [24]. The most common number of tablets prescribed was assumed in approximately 3% of prescriptions were this information was missing or deemed implausible.

### Potential confounders

Potential confounders included those measured at cohort entry including, year of cancer diagnosis and age. Co-morbid conditions have been noted to impact upon survival in cancer patients, including breast cancer [26–28], thus our models adjusted for various comorbities (defined at cohort entry) including cerebrovascular disease, chronic pulmonary disease, congestive heart disease, diabetes, liver disease, myocardial infarction, peptic ulcer disease, peripheral vascular disease, renal disease, identified using a list of Read codes modified for use in the CPRD) [29]. A history of severe mental illness (including schizophrenia-like disorders, bipolar-affective disorders and other non-organic psychoses such as delusional disorder, ‘psychoses not otherwise specified’ and severe depression with psychoses) was identified using Read codes, as used previously [1]. Deprivation data was available from census information, and based on the 2010 Index of Multiple Deprivation (IMD) score of the patient’s postcode. From the NCDR we determined treatment information within 6 months of diagnosis (including surgery, chemotherapy, and radiotherapy). We used CPRD prescription records to identify patients who received hormone therapy treatment (tamoxifen or aromatase inhibitors), and those who had used oral contraceptives (ever use) or hormone replacement therapy (HRT; ever use) prior to diagnosis, as these have been shown to influence breast cancer progression [30, 31]. Finally statin and aspirin use was determined from CPRD and modeled as time-varying covariates, defined as users and non-users, lagged by one-year as outlined above.

### Statistical analyses

Descriptive statistics were used to summarise the characteristics of the cohort. Time-dependent Cox proportional hazards models were used to estimate hazard ratios (HRs) and 95% CIs of breast cancer-specific mortality associated with the post-diagnostic use of antipsychotics compared with non-use. All models were adjusted for the potential confounders measured at cohort entry (statin and aspirin use modelled as time-varying covariates), as outlined above. In secondary analyses we investigated antipsychotics by type (1st generation or 2nd generation), by prolactin elevating and prolactin-sparing antipsychotics (as outlined in Supplementary Table S1) and classifying antipsychotic use as 1st generation antipsychotics only, 2nd generation only or both. Additional analyses investigated individual common antipsychotics including fupentixol, promazine, trifluoperazine, haloperidol, olanzapine, risperidone and quetiapine. Dose-response analysis was conducted to investigate high antipsychotic use compared to low use, using cumulative DDDs. For this time-dependent analysis patients could contribute person-time to both non-user and user groups. Thus antipsychotic users were included in the 1 to 182 DDDs category (low use) until 12 months after they received their 182nd DDD and were considered in the 182+ DDD group thereafter. Similarly, in additional dose-response analysis, cumulative DDDs were categorised into seven pre-defined groups (1–30 DDDs, 30–90 DDDs, 90–180 DDDs, 180–270 DDDs, 270–360 DDDs, 360–540 DDDs and > 540 DDDs).

### Sensitivity analyses investigating confounding by indication

Confounding by indication is an important source of bias in pharmacoepidemiological studies. This bias occurs when the indication for the treatment of interest is also a risk factor for the outcome of interest, thus prognostic variables in the treatment group differ from the control group [32, 33]. A number of analyses were conducted to attempt to compare antipsychotic users to more clinically similar antipsychotic non-users. First, analyses were repeated restricting to patients with a diagnosis of severe mental illness (including schizophrenia-like disorders, bipolar-affective disorders and other non-organic psychoses as outlined previously) at any time prior to breast-cancer diagnosis, including for analyses investigating first and second-generation antipsychotics and for prolactin elevating and sparing antipsychotics. A number of sensitivity analyses were also repeated among the cohort of patients with severe mental illness. Analyses were conducted investigating mortality associated with post-diagnostic prolactin elevating antipsychotics use with post-diagnostic prolactin-sparing antipsychotics as an active cmparator. Analyses were also conducted investigating post-diagnostic antipsychotic use stratified by antipsychotic use in the year prior to diagnosis (i.e. analysis of new-users post-diagnosis).

### Sensitivity and subgroup analyses

A number of additional sensitivity and subgroup analyses were also conducted. Firstly, the primary analysis was repeated expanding the outcome definition of breast cancer-specific mortality to also include deaths in which breast cancer was stated as a secondary underlying cuase of death. Second, the primary analysis was repeated investigating the secondary outcome of all-cause mortality (death due to any cause). Third, analyses were conducted varying the length of the lag period to 6 months, two and 4 years. Fourth, additional analysis used a simplified exposure definition, with antipsychotic use defined in the year post-diagnosis among patients living at least 1 year in our analysis, controlling for immortal time bias without requiring time-varying covariates (as illustrated in Supplementary Fig. S1) [34]. Fifth, as a proxy for breast cancer oestrogen status, analyses were conducted stratifying by use of tamoxifen or aromatase inhibitors (identified from GP prescribing records) within 6 months of breast cancer diagnosis. Further sensitivity analyses were conducted additionally adjusting for stage, smoking and BMI (body mass index). First analyses were conducted using a complete case approach and second utilising multiple imputation [35]. A multiple imputation model for stage used ordinal logistic regression and included age, year, cancer treatment, comorbidities, hormone therapy use, oral contraceptive use, death indicator and the baseline hazard function [35]. Similar imputation models were used for smoking (based upon a multinomial logistic regression) and BMI (based upon a multiple linear regression model). Twenty imputations were conducted and results were combined using Rubin’s rules [35]. A separate analysis was conducted adjusting for stage using complete case restricted to 2997 breast cancer patients from the two cancer registries with the highest rates of available stage (in which stage was 85% complete). Finally, the primary analyses were repeated with antipsychotic use defined in the year prior to diagnosis, not excluding deaths in the first year after diagnosis, (i.e. T0 from breast cancer diaganosis) and adjusting for previous confounders with the exception of cancer treatment (as cancer treatment could be on the causal pathway). All analyses were conducted using Stata/IC (version 14, TX, USA).

## Results

In total, there were 23,695 patients followed for up to 16 years (beyond the one-year lag period) after breast cancer diagnosis (with a median follow-up of 5.5 years). During the follow-up period there were 3061 breast cancer deaths and 848 patients were treated with an antipsychotic medication.

Table 1 includes baseline characteristics by use of antipsychotics defined within the first year post breast cancer diagnosis for the whole cohort and among patients diagnosed with severe mental illness. Overall, compared to non-users, antipsychotic users were more likely to be older, to have a higher deprivation index, to have used aromatase inhibitors but were less likely to undergo surgery or radiotherapy. They were also more likely to have higher staged disease, to have other comorbidities, to have used other medications (excluding HRT) and were less likely to be current smokers. When restricting the cohort to patients with a diagnosis of severe mental illness, patterns in the differences in baseline characteristics remained largely similar, however antipsychotic users were less likely to be within the 70–79 age group and there was no difference in surgery and radiotherapy rates.

**Table 1:** Patient characteristics by antipsychotic use at cohort entry for all patients and by diagnosis of a severe mental illness

	All breast cancer patients		Breast cancer patients with severe mental illness	
	Antipsychotic user ^**a**^	Antipsychotic non-user	Antipsychotic user ^**a**^	Antipsychotic non-user
**Total**	537 (2.3)	23,158 (97.7)	164 (49.1)	170 (50.9)
**Year, n (%)**				
1998–2002	129 (24.0)	5616 (24.3)	36 (22.0)	35 (20.6)
2003–2007	203 (37.8)	8592 (37.1)	60 (36.6)	59 (34.7)
2008–2012	205 (38.2)	8950 (38.6)	68 (41.5)	76 (44.7)
**Age, mean (SD)**	64.7 (14)	62 (14)	62.1 (11.5)	65.4 (14.2)
0–49	93 (17.3)	4927 (21.3)	20 (12.2)	28 (16.5)
50–59	118 (22.0)	5799 (25.0)	52 (31.7)	35 (20.6)
60–69	138 (25.7)	5783 (25.0)	51 (31.1)	42 (24.7)
70–79	188 (35.0)	6649 (28.7)	41 (25.0)	65 (38.2)
**Deprivation, n (%)**				
1 (Least Deprived)	98 (18.2)	5927 (25.6)	31 (18.9)	33 (19.4)
2	121 (22.5)	5974 (25.8)	28 (17.1)	51 (30.0)
3	116 (21.6)	4767 (20.6)	33 (20.1)	34 (20.0)
4	103 (19.2)	3848 (16.6)	32 (19.5)	32 (18.8)
5 (Most Deprived)	99 (18.4)	2633 (11.4)	40 (24.4)	20 (11.8)
**Breast cancer treatment, n (%)**				
Surgery	350 (65.2)	18,947 (81.8)	116 (70.7)	122 (71.8)
Radiotherapy	131 (24.4)	8297 (35.8)	45 (27.4)	46 (27.1)
Chemotherapy	149 (27.7)	6658 (28.8)	37 (22.6)	48 (28.2)
Tamoxifen	211 (39.3)	9902 (42.8)	59 (36.0)	79 (46.5)
Aromatase inhibitors	152 (28.3)	4854 (21.0)	54 (32.9)	45 (26.5)
**Grade, n (%)**				
1	67 (12.5)	3717 (16.1)	25 (15.2)	28 (16.5)
2	235 (43.8)	10,057 (43.4)	73 (44.5)	72 (42.4)
3	140 (26.1)	6907 (29.8)	41 (25.0)	52 (30.6)
4	2 (0.4)	17 (0.1)	0	0
Missing	93 (17.3)	2460 (10.6)	25 (15.2)	18 (10.6)
**Stage, n (%)**				
1	78 (14.5)	4714 (20.4)	31 (18.9)	31 (18.2)
2	89 (16.6)	3844 (16.6)	29 (17.7)	33 (19.4)
3	13 (2.4)	733 (3.2)	*	*
4	17 (3.2)	291 (1.3)	*	*
Missing	340 (63.3)	13,576 (58.6)	95 (57.9)	93 (54.7)
**Comorbidities, n (%)**				
Chronic pulmonary disease	98 (18.2)	3741 (16.2)	25 (15.2)	36 (21.2)
Diabetes	65 (12.1)	1440 (6.2)	16 (9.8)	16 (9.4)
Renal disease	41 (7.6)	1052 (4.5)	28 (17.1)	14 (8.2)
Cerebrovascular disease	24 (4.5)	813 (3.5)	9 (5.5)	10 (5.9)
Peptic ulcer disease	16 (3.0)	492 (2.1)	4 (2.4)	5 (2.9)
Serious mental illness	164 (30.5)	170 (0.7)	164 (100.0)	170 (100.0)
**Statin use, n (%)**	92 (17.1)	3454 (14.9)	38 (23.2)	29 (17.1)
**Aspirin use, n (%)**	97 (18.1)	2901 (12.5)	29 (17.7)	31 (18.2)
**HRT use, n (%)**	151 (28.1)	7571 (32.7)	56 (34.1)	62 (36.5)
**Smoking status, n (%)**				
Current	264 (55.5)	12,705 (61.3)	71 (47.3)	80 (53.0)
Ex	92 (19.3)	4593 (22.2)	28 (18.7)	31 (20.5)
Non-smoker	120 (25.2)	3431 (16.6)	51 (34.0)	40 (26.5)
Missing	61	2429	14	19
**BMI, mean (SD)**	28.1 (6.1)	27.0 (5.5)	29.4 (5.9)	27.1 (6.1)

^a^ Antisychotic use is defined as use of any antipsychotic within one year of breast cancer diagnosis

*Number suppressed due to small cell counts (< 5)

Table 2 presents results from primary analyses. Compared with non-use, antipsychotic use was associated with an increased risk in breast cancer-specific mortality (HR_adj_, 2.25 95%CI 1.90–2.67). In analyses by cumulative DDDs there was no evidence of a dose response relation with high use (DDDs > 182 HR_adj_, 0.93 95%CI 0.56–1.53). In analyses of cumulative DDDs categories, estimates were elevated until 90–180 DDDs (HR_adj_ 2.15 95%CI 1.32–3.49) and decreased thereafter (> 540 DDDs HR_adj_ 0.70 95%CI 0.31–1.59), however the number of events among longer term users was small (Supplementary Table S2). Similar associations were observed for first-generation antipsychotics. HRs were elevated for promazine (HR_adj_, 3.34 95%CI 2.48, 4.50) and haloperidol (HR_adj_, 4.42 95%CI 3.32–5.89). Estimates for second-generation antipsychotics were attenuated towards the null (all second-generation antipsychotics; HR_adj_ 1.26 95%CI 0.91–1.73). Similar associations were observed for those using exclusively first-generation or second-generation antisychotics. In analyses by prolactin elevating activity the highest HRs were observed for prolactin-elevating antipsychotics (prolactin elevating HR_adj_, 2.27 95%CI 1.90–2.72; prolactin-sparing HR_adj_, 1.27 95%CI 0.87–1.87) and there was no evidence of a dose response relationships for either class.

**Table 2:** Crude and adjusted hazard ratios for the association between the use of antipsychotics and breast cancer-specific mortality

	Users			Non-Users			Unadjusted HR (95% CI)	Adjusted ^**a**^ HR (95% CI)
	N	Person years	Cancer deaths	N	Person years	Cancer deaths		
**All antipsychotics**	848	3190	165	22,847	123,106	2896	2.42 (2.07–2.83)	2.25 (1.90–2.67)
1–182 DDDs v non-user	638	2271	148	22,847	123,106	2896	2.95 (2.50–3.48)	2.56 (2.15–3.04)
182+ DDDs v non-user	210	919	17	22,847	123,106	2896	0.95 (0.59–1.53)	0.93 (0.56–1.53)
**1st generation antipsychotics**	558	2288	124	23,137	124,008	2937	2.62 (2.19–3.13)	2.41 (2.00–2.91)
1–182 DDDs v non-user	462	1787	115	23,137	124,008	2937	3.04 (2.52–3.67)	2.69 (2.22–3.25)
182+ DDDs v non-user	96	501	9	23,137	124,008	2937	0.94 (0.49–1.80)	0.92 (0.47–1.81)
Fupentixol	108	507	14	23,587	125,789	3047	1.36 (0.81–2.30)	1.45 (0.85–2.45)
Promazine	162	447	45	23,533	125,849	3016	4.82 (3.59–6.47)	3.34 (2.48–4.50)
Trifluoperazine	71	432	10	23,624	125,865	3051	1.11 (0.60–2.07)	1.12 (0.60–2.10)
Haloperidol	142	400	50	23,553	125,896	3011	5.71 (4.32–7.56)	4.42 (3.32–5.89)
**2nd generation antipsychotics**	377	1264	45	23,318	125,032	3016	1.60 (1.19–2.15)	1.26 (0.91–1.73)
1–182 DDDs v non-user	251	810	37	23,318	125,032	3016	1.96 (1.41–2.71)	1.48 (1.05–2.08)
182+ DDDs v non-user	126	454	8	23,318	125,032	3016	0.87 (0.43–1.73)	0.70 (0.34–1.44)
Olanzapine	138	546	18	23,557	125,750	3043	1.57 (0.99–2.50)	1.25 (0.76–2.03)
Risperidone	130	463	17	23,565	125,833	3044	1.67 (1.04–2.69)	1.27 (0.78–2.07)
Quetiapine	133	360	12	23,562	125,937	3049	1.55 (0.88–2.73)	1.20 (0.67–2.13)
**1st generation antipsychotics only**	471	1926	120	22,847	123,106	2896	2.94 (2.44, 3.53)	2.75 (2.28, 3.32)
**2nd generation antipsychotics only**	290	902	41	22,847	123,106	2896	1.95 (1.43, 2.65)	1.67 (1.21, 2.32)
**1st and 2nd generation use**	87	362	4	22,847	123,106	2896	0.64 (0.24, 1.69)	0.57 (0.21, 1.54)
**Prolactin elevating antipsychotics** ^**b**^	668	2644	139	23,027	123,652	2922	2.50 (2.11–2.96)	2.27 (1.90–2.72)
1–182 DDDs v non-user	568	2127	130	23,027	123,652	2922	2.84 (2.38–3.39)	2.47 (2.06–2.96)
182+ DDDs v non-user	100	518	9	23,027	123,652	2922	0.91 (0.47–1.76)	0.92 (0.47–1.81)
**Prolactin-sparing antipsychotics** ^**c**^	267	869	30	23,428	125,427	3031	1.58 (1.10–2.27)	1.27 (0.87–1.87)
1–182 DDDs v non-user	144	420	22	23,428	125,427	3031	2.24 (1.47–3.40)	1.75 (1.14–2.69)
182+ DDDs v non-user	123	448	8	23,428	125,427	3031	0.88 (0.44–1.75)	0.70 (0.34–1.44)

^a^ Model contains age, year of diagnosis, treatment within 6 months (separate variables for radiootherapy, chemotherapty, surgery, tamoxifen and aromatase inhibitor use), comorbidities (prior to diagnosis including serious mental illness, chronic pulmonary disease, diabetes, renal disease, cerebrovascular disease, peripheral vascular disease, myocardial infarction, peptic ulcer disease and liver disease), hormonal medication use (oral contraceptive and hormone replacement therapy, prior to diagnosis), other medication use (statin and aspirin as time varying covariates) and deprivation (in fifths)

^b^ Prolactin elevating antipsychotics included chlorpromazine,flupentixol,fluphenazine, haloperidol, pericyazine, perphenazine, pimozide, pipotiazine, promazine, trifluoperazine, zuclopenthixol, amisulpride, risperidone and sulpiride

^c^ Prolactin non-elevating antipsychotics included aripiprazole, olanzapine, quetiapine and sertindole

### Analyses investigating confounding by indication

Table 3 presents analyses restricting the cohort to patients with a diagnosis of severe mental illness. Overall, compared to non-use, antipsychotic use was not associated with breast cancer-specific mortality (HR_adj_, 1.11 95%CI 0.58–2.14). Null associations were also observed for 1st and 2nd generation antipsychotics (HR_adj_, 0.95 95%CI 0.44–2.04; HR_adj_, 1.10 95%CI 0.55–3.28, respectively), as well as for those using exclusively first- or second-generation antipsychotics. Likewise, no associations were observed for prolactin elevating (HR_adj_ 0.86 95%CI 0.44–1.68) and prolactin-sparing antipsychotics (HR_adj_, 1.19 95%CI 0.58–2.44), with no evidence of a dose response relation overall, or by antipsychotic grouping. Sensitivity and subgroup analyses conducted among patients with a severe mental illness diagnosis prior to breast cancer diagnosis also revealed null associations (Table 4). In sensitivity analyses comparing prolactin elevating antipsychotic use to prolactin-sparing antipsychotic use, HRs were attenuated and no longer remaining statistically significant when compared to prolactin-sparing only (HR_adj_, 1.22 95%CI 0.80–1.86). Likewise, estimates were attenuated in analyses stratifying by prior antipsychotic use with null associations observed among patients using antipsychotics in the year prior to diagnosis (Table 4).

**Table 3:** Crude and adjusted hazard ratios for the association between the use of antipsychotics and breast cancer-specific mortality in patients with severe mental illness

	Users			Non-Users			Unadjusted HR (95% CI)	Adjusted^**a**^ HR (95% CI)
	N	Person years	Cancer deaths	N	Person years	Cancer deaths		
**All antipsychotics**	188	898	25	146	658	23	0.97 (0.54–1.74)	1.11 (0.58–2.14)
1–182 DDDs v non-user	79	382	14	146	658	23	1.11 (0.57, 2.17)	1.37 (0.65, 2.88)
182+ DDDs v non-user	109	516	11	146	658	23	0.82 (0.39, 1.74)	0.87 (0.38, 1.98)
**1st generation antipsychotics**	97	511	11	237	1044	37	0.75 (0.38–1.50)	0.95 (0.44–2.04)
1–182 DDDs v non-user	47	234	6	237	1044	37	0.81 (0.34, 1.92)	0.99 (0.38, 2.58)
182+ DDDs v non-user	50	277	5	237	1044	37	0.69 (0.27, 1.81)	0.90 (0.31, 2.58)
**2nd generation antipsychotics**	130	565	16	204	991	32	1.08 (0.59–1.99)	1.10 (0.55–2.18)
1–182 DDDs v non-user	60	288	10	204	991	32	1.24 (0.61, 2.55)	1.44 (0.67, 3.09)
182+ DDDs v non-user	70	276	6	204	991	32	0.88 (0.36, 2.14)	0.72 (0.27, 1.93)
**1st generation antipsychotics only**	58	333	9	146	658	23	0.87 (0.40, 1.90)	1.07 (0.45, 2.56)
**2nd generation antipsychotics only**	91	387	14	146	658	23	1.16 (0.59, 2.27)	1.20 (0.55, 2.61)
**1st and 2nd generation use**	39	178	2	146	658	23	0.56 (0.13, 2.51)	0.80 (0.17, 3.79)
**Prolactin elevating antipsychotics**^**b**^	131	681	15	203	875	33	0.73 (0.39–1.36)	0.86 (0.44–1.68)
1–182 DDDs v non-user	80	395	10	203	875	33	0.77 (0.38, 1.57)	0.87 (0.40, 1.89)
182+ DDDs v non-user	51	286	5	203	875	33	0.65 (0.25, 1.73)	0.83 (0.29, 2.40)
**Prolactin non-elevating antipsychotics**^**c**^	101	417	13	233	1138	35	1.30 (0.68–2.49)	1.19 (0.58–2.44)
1–182 DDDs v non-user	32	143	7	233	1138	35	2.00 (0.88, 4.55)	2.00 (0.85, 4.69)
182+ DDDs v non-user	69	275	6	233	1138	35	0.92 (0.38, 2.22)	0.73 (0.28, 1.93)

^a^Model contains age, year of diagnosis, treatment within 6 months (separate variables for radiootherapy, chemotherapty, surgery, tamoxifen and aromatase inhibitor use), comorbidities (prior to diagnosis including chronic pulmonary disease, diabetes, renal disease, cerebrovascular disease, peripheral vascular disease, myocardial infarction, peptic ulcer disease and liver disease), hormonal medication use (oral contraceptive and hormone replacement therapy, prior to diagnosis), other medication use (statin and aspirin as time varying covariates) and deprivation (in fifths)

^b^Prolactin elevating antipsychotics included chlorpromazine,flupentixol,fluphenazine, haloperidol, pericyazine, perphenazine, pimozide, pipotiazine, promazine, trifluoperazine, zuclopenthixol, amisulpride, risperidone and sulpiride

^c^Prolactin non-elevating antipsychotics included aripiprazole, olanzapine, quetiapine and sertindole

**Table 4:** **Sensitivity and subgroup analysis for the association between antipsychotic use and breast cancer mortality**

	N	Person years	Cancer deaths	All antipsychotics	Prolactin elevating antipsychotics	Prolactin –sparing antipsychotics
				Adjusted HR^**a**^	Adjusted HR^**a**^	Adjusted HR^**a**^
**All breast cancer patients**						
**Main analysis**	23,695	126,296	3061	2.25 (1.90–2.67)	2.27 (1.90–2.72)	1.27 (0.87–1.87)
**Outcome definition**						
All-cause mortality	23,695	126,296	6268	2.07 (1.84–2.31)	2.02 (1.78–2.28)	1.68 (1.36–2.08)
Breast cancer on death certificate	23,695	126,296	3726	2.19 (1.88–2.54)	2.13 (1.81–2.50)	1.54 (1.14–2.10)
**Exposure definition**						
Year after diagnosis^b^	23,695	126,296	3061	1.70 (1.38–2.09)	1.63 (1.30–2.04)	1.43 (0.94–2.18)
6 month lag	24,973	138,467	3442	2.76 (2.40–3.18)	2.91 (2.51–3.37)	1.41 (1.01–1.95)
2 year lag	21,097	103,895	2278	1.47 (1.15–1.88)	1.39 (1.06–1.81)	1.30 (0.81–2.10)
4 year lag	15,508	67,078	1190	1.17 (0.78, 1.73)	1.12 (0.74, 1.71)	0.92 (0.39, 2.15)
Prolactin elevating versus sparing antipsychotics^c1^		3190	165	–	1.22 (0.80–1.86)	1.00
Prolactin elevating versus sparing antipsychotics^c2^		3190	165	–	1.64 (1.10–2.46)	1.00
**Stratified analysis**						
Tamoxifen or AI use^d^	14,657	80,780	1707	2.19 (1.76–2.71)	2.09 (1.65–2.65)	1.67 (1.09–2.57)
No hormonal therapy use^d^	9038	45,516	1354	2.28 (1.73–2.99)	2.46 (1.86–3.25)	0.67 (0.30–1.52)
Prior antipsychotic use^e^	389	1828	65	0.97 (0.51–1.84)	0.77 (0.45–1.33)	1.27 (0.66–2.42)
No prior antipsychotic use^e^	23,306	124,468	2996	2.83 (2.34–3.42)	3.08 (2.53–3.76)	1.10 (0.63–1.91)
**Additional adjustment**						
Stage adjusted using CC^f^	9778	51,043	1080	2.30 (1.70–3.10)	2.46 (1.80–3.36)	0.98 (0.47–2.06)
Stage adjusted using MI^g^	23,686	126,258	3059	2.27 (1.89–2.71)	2.31 (1.91–2.80)	1.22 (0.82–1.81)
Stage (CC in cancer registries highest availability^h^)	2612	13,311	338	2.40 (1.29, 4.47)	2.83 (1.53, 5.26)	0.91 (0.23, 3.52)
Smoking and BMI adjusted using CC^f^	18,135	95,342	2167	2.42 (1.97–2.96)	2.53 (2.05–3.14)	1.21 (0.73–2.01)
Smoking and BMI adjusted using MI^g^	23,686	126,258	3059	2.24 (1.89–2.65)	2.25 (1.88–2.70)	1.27 (0.86–1.87)
**Patients with severe mental illness prior to diagnosis**						
**Main analysis**	334	1556	48	1.11 (0.58–2.14)	0.86 (0.44–1.68)	1.19 (0.58–2.44)
**All-cause mortality**	334	1556	121	1.13 (0.75–1.71)	1.13 (0.76–1.68)	1.12 (0.70–1.77)
**6 month lag**	364	1730	60	1.17 (0.66–2.08)	1.07 (0.60–1.92)	1.22 (0.64–2.30)
**2 year lag**	288	1245	35	1.60 (0.72, 3.58)	1.17 (0.53, 2.58)	1.74 (0.73, 4.17)
**4 year lag**	187	771	14	2.04 (0.50, 8.34)	1.00 (0.27, 3.66)	2.65 (0.67, 10.47)
**Tamoxifen or AI use**^**d**^	230	1109	31	1.02 (0.41–2.56)	0.86 (0.36–2.06)	1.27 (0.47–3.40)
**Stage adjusted using MI**^**g**^	334	1556	48	1.16 (0.58–2.32)	1.02 (0.49–2.10)	1.01 (0.46–2.20)

^a^Model contains age, year of diagnosis, treatment within 6 months (separate variables for radiootherapy, chemotherapty, surgery, tamoxifen and aromatase inhibitor use), comorbidities (prior to diagnosis including serious mental illness (except when analysis restricted to patients with severe mental illness prior to diagnosis), chronic pulmonary disease, diabetes, renal disease, cerebrovascular disease, peripheral vascular disease, myocardial infarction, peptic ulcer disease and liver disease), hormonal medication use (oral contraceptive and hormone replacement therapy, prior to diagnosis), other medication use (statin and aspirin as time varying covariates) and deprivation (in fifths)

^b^ Anti-psychotic use based upon use in the year after breast cancer diagnosis adjusting for variables in ^a^

c1 Prolactin elevating antipsychotics versus prolactin non-elevating antipsychotics (only prolactin elevating or both prolactin elevating and non-elevating, versus only prolactin non-elevating)

c2 Prolactin elevating antipsychotics versus prolactin non-elevating antipsychotics (only prolactin elevating, versus both prolactin elevating and non-elevating or only prolactin non-elevating)

^d^ Stratified based upon hormonal therapy use (AI or tamoxifen) in the 6 months after diagnosis

^e^ Stratified based upon use of any antipsychotic medication in the year prior to diagnosis

^f^ Complete case analysis, adjusted analysis additionally adjusted for exposure (stage or smoking and BMI)

^g^ Using multiple imputation to impute missing exposure (stage or smoking and BMI)

^h^ Complete case analysis additionally adjusting for stage restricted to two cancer registries in which stage was 85% complete

### Subgroup and sensitivity analyses

Overall, sensitivity analyses remained largely similar to the primary analyses, with similar associations observed for all-cause mortality and with breast cancer listed at any position on the death certificate (Table 4). HRs remained elevated when defining antipsychotic use in the year after diagnosis, as well as in analyses applying a 6-month lag, 2-year lag. Analysis with a 4-year lag attenuated estimates towards the null (HR_adj_1.17 95%CI 0.78, 1.73). Results remained similar for all antipsychotics and prolactin-elevating antipsychotics when stratifying by receipt of hormonal therapy; however, estimates for prolactin-sparing antipsychotics were more marked for hormonal therapy users (HR_adj_, 1.67 95%CI 1.09–2.47) compared with non-users (HR_adj_, 0.67 95%CI 0.30–1.52). Additional adjustment for stage and BMI and smoking revealed largely similar estimates. Estimates were slightly attenuated in analyses of antipsychotic use in the year prior to diagnosis (HR_adj_, 1.52 95%CI 1.21–1.91)[presented in Supplementary Table S3].

## Discussion

In this large population-based study, we observed increases in breast cancer-specific mortality among patients using antipsychotics after diagnosis, with marked associations observed for prolactin-elevating antipsychotics. However, these associations did not appear to follow a dose-response pattern. Importantly, analyses restricting the cohort to patients with a history of severe mental illness and analyses comparing prolactin elevating and prolactin-sparing antipsychotics all revealed null associations. Thus, taken together these results appear to suggest that the associations observed are a result of confounding by indication i.e. that patients with severe mental illness are at increased risk of breast cancer-specific mortality and that these patients are more likely to receive antipsychotics.

To the best of our knowledge, this is the first observational study to investigate the association between antipsychotic use and breast cancer survival. It has previously been suggested that antipsychotics, via their effects on prolactin levels, may influence breast cancer prognosis. Prolactin receptors have been observed in breast cancer tissue [6] and a number of studies have reported proliferative and metastatic effects of prolactin in vitro [36–38]. In breast cancer patients, high prolactin levels pre-treatment have also been associated with increased treatment failure, recurrence and decreased survival [7–9, 39, 40]. Indeed, in this study, while we observed higher risks of breast cancer mortality for prolactin-elevating antipsychotics than prolactin-sparing in the overall cohort (HR_adj_, 2.27 95%CI 1.90–2.72; HR_adj_,1.27 95%CI 0.87–1.87, respectively), this failed to follow a dose-response pattern and associations were attenuated when comparing prolactin-elevating to prolactin-sparing antipsychotics. Nonetheless, evidence regarding the role of prolactin on breast cancer carcinogenesis remains conflicting [19, 41]. Additionally, the development of prolactin receptor blocking agents have so far proved ineffective for breast cancer treatment [42, 43].

While we cannot rule out a causal relationship between breast cancer-specific mortality and antipsychotic use, these findings should be interpreted with caution as they are likely vulnerable to confounding by indication. A number of studies have reported that patients with severe mental illness, including schizophrenia and bipolar disorder have an increased risk of breast cancer and may have up to a 3-fold increased risk of breast cancer mortality [44–46]. Women with severe mental illness may experience delays in breast cancer detection due to a lower awareness of breast cancer symptoms and low uptake of mammography and as such often present with higher stage disease [46–48]. However, in our study additionally adjusting for stage revealed similar results. Additionally, patients with breast cancer and severe mental illness also have higher rates of smoking, other adverse lifestyle behaviours, higher morbidity and are less likely to receive appropriate cancer care or often experience delays in cancer treatment and poor adherence, thus all contributing to decreased survival [47, 49–52]. Indeed, in our study antipsychotic users were less likely to receive surgery and radiotherapy, while restricting the cohort to patients with severe mental illness revealed similar rates of surgery and radiotherapy among users and non-users (although rates of chemotherapy were higher in non-users). Moreover, in analyses in patients with a history of severe mental illness results where attenuated towards the null, including for prolactin-elevating antipsychotics (HR_adj_, 0.86 95%CI 0.44, 1.68), suggesting our overall results are likely influenced by confounding by indication. These discrepancies in results observed for the overall cohort and when restricting to patients with severe mental illness, and comparing prolactin elevating antipsycotics to prolacting–sparing antipsychotics provide a clear example of the importance of accounting for confounding by indication in pharmacoepidemiolgical studies in cancer patients. However, while the potential association between prolactin-elevating antipsychotics and breast cancer survival requires further exploration these results when restricting to patients with similar diagnoses should provide some reassurance for clinicians around the use of antipsychotics in breast cancer patients, in whom psychiatric disorders are often undertreated [19].

### Strengths and limitations

This study had a number of strengths. Firstly, this was a large population-based study, utilizing high quality data including registry confirmed breast cancer and had a long follow-up period of up to 16 years (beyond the 1 year post-cohort lag period). Linkage to the ONS death registration data allowed for robust verification of death, and facilitated breast cancer-specific analysis which should be more sensitive to small changes in disease-specific mortality and less susceptible to confounding by indication than all-cause mortality [53, 54]. Furthermore, we used a time varying exposure definition that eliminated immortal time bias while also account for latency considerations. Finally, the use of the CPRD and NCDR allowed us to adjust for several potentially important confounders including for example age, comorbidities and smoking status.

However, this study also had a number of limitations. First, although we were able to adjust for a number of important confounders we also lacked information on other potential confounders such as ethnicity or dietary factors. Furthermore, tumour stage was missing for a proportion of our cohort and thus omitted from our main analyses. Reassuringly, our results remained consistent when adjusting for stage using a range of approaches, for instance, using multiple imputation for missing stage and in complete case analyses of stage restricted to cancer registries with stage availability of over 85%. We also lacked information on hormone receptor status however we were able to adjust for tamoxifen and aromatase inhibitor use as a proxy for oestrogen status. While we had detailed information on antipsychotic drug use from GP prescribing data, including type, strength and quantity, this reflects those written by general practitioners, rather than dispensing information, thus misclassification of exposure is possible if patients did not adhere to the treatment regimen or received prescriptions from specialists. However we were able to conduct analyses by cumulative DDDs (e.g. > 182 DDDs) for whom non-compliance is less of a concern. Additionally, although previous studies have reported overall high levels of diagnostic validity in CPRD, to the best of our knowledge no previous study has investigated the validity of psychosis or bipolar disorder diagnoses in CPRD [55, 56]. Reassuringly, a previous study in UK general practice did report high accuracy and completeness of psychosis diagnoses however misclassification of these cannot be ruled out [57]. Finally, while antipsychotics are not available over the counter in the UK, which negates exposure misclassification due to over-the-counter use, antipsychotic prescriptions from secondary care are not captured within the CPRD so some exposure misclassification is possible.

## Conclusion

This was the first study to date to examine the association between post-diagnostic antipsychotic use and survival in patients with breast cancer. While we observed increases in breast cancer-specific mortality, the lack of a dose response relation, and the null associations observed in patients with severe mental illness, suggest the observed association is likely a result of confounding by indication. This highlights the importance of controlling for this bias in studies of drug effects in cancer patients and should provide some reassurance to clinicans on the use of antipsychotic medicatons in women diagnosed with breast cancer.

## Supplementary information

**Additional file 1 Table S1.** Classification of Antipsychotics. **Table S2.** Crude and adjusted hazard ratios for the association between the use of antipsychotics and breast cancer-specific mortality by cumulative DDDs. **Table S3.** Crude and adjusted hazard ratios for the association between the use of antipsychotics in the year prior to diagnosis and breast cancer-specific mortality. **Fig. S1.** Figure illustrating exposure definitions for primary and sensitivity analyses.

## Data Availability

The data that support the findings of this study are available from CPRD. Restrictions apply to the availability of these data, which were used under license for the current study and so are not publicaly available. Data are however available from the authors upon reasonable request and permission from the Clinical Practice Research Datalink.

## Abbreviations

AI: Aromatase inhibitors
BBB: Blood brain barrier
BMI: Body mass index
CC: Complete case
CI: Confidence interval
CPRD: Clinical Practice Research Datalink
D2R: Dopamine receptor D_2_
DDD: Defined daily dose
HR: Hazard ratio
HRT: Hormone replacement therapy
IMD: Inedx of Multiple Deprivation
ICD: International Statistical Classificaion of Diseases and Related Health Problems
MI: Multiple imputation
NCDR: National Cancer Data repository
ONS: Office of National Statistics
SD: Standard deviation
UK: United Kingdom
USA: United States of America
