# Supplementary material for: Post-diagnostic antipsychotic use and cancer mortality: a population based cohort study
Source: BMC Cancer. 2020 Aug 24;20:804. doi: 10.1186/s12885-020-07320-3 (PMC7446212; doi:10.1186/s12885-020-07320-3)
Supplement: Supplementary file 1 — Additional file 1 Table S1. Classification of Antipsychotics. Table S2. Crude and adjusted hazard ratios for the association between the use of antipsychotics and breast cancer-specific mortality by cumulative DDDs. Table S3. Crude and adjusted hazard ratios for the association between the use of antipsychotics in the year prior to diagnosis and breast cancer-specific mortality. Fig. S1. Figure illustrating exposure definitions for primary and sensitivity analyses. [file 12885_2020_7320_MOESM1_ESM.docx]

**Supplementary Material**

| **Table S1** | Classification of Antipsychotics |
| --- | --- |
| **Table S2** | Crude and adjusted hazard ratios for the association between the use of antipsychotics and breast cancer-specific mortality by cumulative DDDs |
| **Table S3** | Crude and adjusted hazard ratios for the association between the use of antipsychotics in the year prior to diagnosis and breast cancer-specific mortality |
| **Figure S1** | Figure illustrating exposure definitions for primary and sensitivity analyses |
|  |  |
|  |  |
|  |  |

**Table S1 Classification of Included Antipsychotics**

|  | **First generation Antipsychotics** | **Second generation Antipsychotics** |
| --- | --- | --- |
| **Prolactin-elevating Antipsychotics** |  |  |
|  | Chlorpromazine  Flupentixol  Fluphenazine  Haloperidol  Pericyazine  Perphenazine  Sulpiride  Pimozide  Pipotiazine  Promazine  Trifluoperazine  Zuclopenthixol | Risperidone  Amisulpride |
| **Prolactin-sparing Antipsychotics** |  |  |
|  |  | Aripiprazole  Olanzapine  Quetiapine,  Sertindole. |

**Table S2 Crude and adjusted hazard ratios for the association between the use of antipsychotics and breast cancer-specific mortality by cumulative DDDs**

|  | **Users** | | |  | **Non-Users** | | | **Unadjusted HR (95% CI)** | **Adjusted ^a^**  **HR (95% CI)** |
| --- | --- | --- | --- | --- | --- | --- | --- | --- | --- |
|  | **N** | **Person years** | **Cancer deaths** |  | **N** | **Person years** | **Cancer deaths** |  |  |
| **All antipsychotics** |  |  |  |  |  |  |  |  |  |
| 1-30 DDDs v non-user | 448 | 1564 | 108 |  | 22,847 | 123,106 | 2,896 | 3.19 (2.63- 3.86) | 2.72 (2.24-3.32) |
| 30-90 DDDs v non-user | 110 | 430 | 23 |  | 22,847 | 123,106 | 2,896 | 2.30 (1.52-3.46) | 2.17 (1.43-3.29) |
| 90-180 DDDs v non-user | 79 | 275 | 17 |  | 22,847 | 123,106 | 2,896 | 2.72 (1.69-4.38) | 2.15 (1.32-3.49) |
| 180-270 DDDs v non-user | 41 | 159 | 5 |  | 22,847 | 123,106 | 2,896 | 1.44 (0.60-3.47) | 1.20 (0.50-2.91) |
| 270-360 DDDs v non-user | 24 | 110 | 3 |  | 22,847 | 123,106 | 2,896 | 1.31 (0.42-4.06) | 1.19 (0.38-3.73) |
| 360-540 DDDs v non-user | 34 | 137 | 3 |  | 22,847 | 123,106 | 2,896 | 1.00 (0.32-3.11) | 0.85 (0.27-2.67) |
| 540+ DDDs v non-user | 112 | 515 | 6 |  | 22,847 | 123,106 | 2,896 | 0.65 (0.29-1.44) | 0.70 (0.31-1.59) |
|  |  |  |  |  |  |  |  |  |  |

^a^ Model contains age, year of diagnosis, treatment within 6 months (separate variables for radiootherapy, chemotherapty, surgery, tamoxifen and aromatase inhibitor use), comorbidities (prior to diagnosis including serious mental illness, chronic pulmonary disease, diabetes, renal disease, cerebrovascular disease, peripheral vascular disease, myocardial infarction, peptic ulcer disease and liver disease), hormonal medication use (oral contraceptive and hormone replacement therapy, prior to diagnosis), other medication use (statin and aspirin as time varying covariates) and deprivation (in fifths).

**Table S3 Crude and adjusted hazard ratios for the association between the use of antipsychotics in the year prior to diagnosis and breast cancer-specific mortality**

|  | **Users^e^** | | |  | **Non-Users** | | | **Unadjusted HR** | **Adjusted HR^a^** |
| --- | --- | --- | --- | --- | --- | --- | --- | --- | --- |
|  | **N** | **PY** | **Deaths** |  | **N** | **Person-years** | **Deaths** |  |  |
|  |  |  |  |  |  |  |  |  |  |
| All antipsychotics | 457 | 2043 | 98 |  | 23,995 | 135,904 | 3,535 | 1.81 (1.48-2.21) | 1.52 (1.21-1.91) |
|  |  |  |  |  |  |  |  |  |  |
| **1^st^ gen antipsychotics** | 277 | 1440 | 58 |  | 24,175 | 136,507 | 3,575 | 1.58 (1.22-2.05) | 1.24 (0.94-1.64) |
|  |  |  |  |  |  |  |  |  |  |
| Fupentixol | 54 | 323 | 9 |  | 24,398 | 137,624 | 3,624 | 1.11 (0.58-2.13) | 0.94 (0.49-1.82) |
| Promazine | 22 | 62 | 5 |  | 24,430 | 137,884 | 3,628 | 2.68 (1.12-6.45) | 1.95 (0.81-4.70) |
| Trifluoperazine | 60 | 373 | 9 |  | 24,392 | 137,573 | 3,624 | 0.96 (0.50-1.85) | 0.77 (0.40-1.50) |
| Haloperidol | 54 | 181 | 21 |  | 24,398 | 137,765 | 3,612 | 4.38 (2.85-6.73) | 3.07 (1.98-4.77) |
|  |  |  |  |  |  |  |  |  |  |
| **2^nd^ gen antipsychotics** | 204 | 698 | 47 |  | 24,248 | 137,248 | 3,586 | 2.29 (1.72-3.06) | 1.95 (1.42-2.69) |
|  |  |  |  |  |  |  |  |  |  |
| Olanzapine | 70 | 269 | 11 |  | 24,382 | 137,678 | 3,622 | 1.42 (0.79-2.57) | 1.27 (0.69-2.36) |
| Risperidone | 67 | 242 | 21 |  | 24,385 | 137,705 | 3,612 | 2.98 (1.94-4.58) | 2.03 (1.30-3.16) |
| Quetiapine | 56 | 160 | 15 |  | 24,396 | 137,786 | 3,618 | 3.05 (1.84-5.07) | 2.65 (1.57-4.48) |
| Amisulpride | 14 | 40 | 2 |  | 24,438 | 137,906 | 3,631 | 1.64 (0.41-6.57) | 0.89 (0.22-3.59) |
| Aripiprazole | 9 | 35 | 2 |  | 24,443 | 137,912 | 3,631 | 1.91 (0.48-7.64) | 2.05 (0.51-8.31) |
|  |  |  |  |  |  |  |  |  |  |
| **Prolactin elevating antipsychotics** | 347 | 1678 | 77 |  | 24,105 | 136,269 | 3,556 | 1.77 (1.41-2.22) | 1.39 (1.08-1.77) |
|  |  |  |  |  |  |  |  |  |  |
| **Prolactin non-elevating antipsychotics** | 130 | 437 | 28 |  | 24,322 | 137,509 | 3,605 | 2.16 (1.49-3.13) | 2.04 (1.36-3.06) |

^a^ Adjusting for age-year of diagnosis-comorbidities (prior to diagnosis including serious mental illness-chronic pulmonary disease-diabetes-renal disease-cerebrovascular disease-congestive heart disease-peripheral vascular disease-myocardial infarction-peptic ulcer disease and liver disease) and other medication use (statin and aspirin as time varying covariates).

**Figure S1 Figure illustrating exposure definitions for primary and sensitivity analyses.**

First antipsychotic Non-user time

Breast Start of Cancer death/

cancer follow-up censoring

diagnosis (1 year)

Non-user time User time

Sensitivity analysis: antipsychotic TVC with 2-year lag.

*2-year lag*

Non-user time User time

*1-year lag*

Breast Start of First antipsychotic Cancer death/

cancer follow-up censoring

diagnosis (1 year)

Main analysis: antipsychotic time-varying covariate (TVC).

Breast Start of First antipsychotic Cancer death/

cancer follow-up censoring

diagnosis (2 years)

First antipsychotic User time

Sensitivity analysis: antipsychotic use in the first year after diagnosis.

Breast Start of Cancer death/

cancer follow-up censoring

diagnosis (1 year)
